# The Science and Practice of Carcinogen Identification and Evaluation

**DOI:** 10.1289/ehp.6950

**Published:** 2004-06-03

**Authors:** Vincent James Cogliano, Robert A. Baan, Kurt Straif, Yann Grosse, Marie Béatrice Secretan, Fatiha El Ghissassi, Paul Kleihues

**Affiliations:** International Agency for Research on Cancer, Lyon, France

**Keywords:** carcinogen, carcinogen identification, conflict of interests, hazard identification, *IARC Monographs*

## Abstract

Several national and international health agencies have established programs with the aim of identifying agents and exposures that cause cancer in humans. Carcinogen identification is an activity grounded in the scientific evaluation of the results of human epidemiologic studies, long-term bioassays in experimental animals, and other data relevant to an evaluation of carcinogenicity and its mechanisms. In this commentary, after a brief discussion of the science basis common to the evaluation of carcinogens across different programs, we discuss in more detail the principles and procedures currently used by the IARC Monographs program.

The global burden of cancer continues to increase. There were an estimated 10.1 million new cases, 6.2 million deaths, and 22.4 million persons living with cancer in the year 2000 (Kleihues and Stewart 2003). This represents an increase of 19% in incidence and 18% in mortality since 1990. Given current trends in smoking prevalence and other factors, the annual number of new cases is estimated to reach 15 million by 2020. It is possible to prevent at least one-third of these new cases through better use of existing knowledge.

Understanding how cancer develops creates opportunities for cancer prevention or early detection. An important part of this effort is to identify the agents and exposures that cause cancer. Several national and international health agencies have established carcinogen identification programs that provide a scientific basis for governmental and private efforts to control cancer by reducing exposure to known and suspected human carcinogens.

The *IARC Monographs on the Evaluation of Carcinogenic Risks to Humans* are published by the International Agency for Research on Cancer (IARC) of the World Health Organization (WHO). Each *IARC Monograph* represents the consensus of an international working group of expert scientists. The *IARC Monographs* include a critical review of the pertinent peer-reviewed scientific literature as the basis for an evaluation of the weight of the evidence that an agent may be carcinogenic to humans. Published continuously since 1972, the scope of the *IARC Monographs* has expanded beyond chemicals to include complex mixtures, occupational exposures, lifestyle factors, physical and biologic agents, and other potentially carcinogenic exposures. After publication of *IARC Monograph* volume 87, expected in 2004 or 2005, nearly 900 agents, mixtures, and exposures will have been evaluated. Among these, 91 have been characterized as carcinogenic to humans, 67 as probably carcinogenic to humans, and 240 as possibly carcinogenic to humans.

The U.S. National Toxicology Program (NTP; Research Triangle Park, NC, USA) publishes the *Report on Carcinogens*, which identifies and discusses substances that may pose a carcinogenic hazard to human health and to which a significant number of persons residing in the United States are exposed. Mandated in 1978 by an act of the U.S. Congress, the *Report on Carcinogens* lists agents as either “known to be a human carcinogen” or “reasonably anticipated to be a human carcinogen.” One nongovernmental and two federal scientific committees review the nominations for listing in or delisting from the *Report on Carcinogens*. The director of the National Toxicology Program reviews the three groups’ recommendations and all public comments before the Secretary of Health and Human Services reviews and approves the *Report on Carcinogens* (NTP 2002).

The U.S. Environmental Protection Agency (EPA) assesses the health hazards of chemical contaminants present in the environment. These assessments cover cancer and adverse effects other than cancer. The hazard assessments are coupled with dose–response assessments that the U.S. EPA uses in its regulatory and information programs. The principles that the U.S. EPA uses in its cancer assessments are discussed in an evolving series of guidelines (U.S. EPA 1986 U.S. EPA 1999 U.S. EPA 2003). Chemical assessments are developed through a process that includes a toxicologic review of the pertinent scientific literature written by U.S. EPA scientists or contractors, internal and external peer reviews, and an internal consensus review (U.S. EPA 2004).

The California Environmental Protection Agency (Cal/EPA) maintains a list of “chemicals known to the state to cause cancer” under Proposition 65 (Cal/EPA 2004), a 1986 ballot initiative enacted to protect citizens from chemicals known to cause cancer, birth defects, or other reproductive harm and to inform citizens about exposures to such chemicals. A chemical is listed if an independent committee of scientists and health professionals finds that the chemical has been clearly shown to cause cancer, if an authoritative body (currently the U.S. EPA, the U.S. Food and Drug Administration, the National Institute for Occupational Safety and Health, the NTP, and IARC) has identified it as causing cancer, or if a California or U.S. government agency requires that it be labeled or identified as causing cancer (Cal/EPA 2004).

These programs have developed the following descriptors:

**IARC (IARC 2004)**

- Carcinogenic to humans (group 1)
- Probably carcinogenic to humans (group 2A)
- Possibly carcinogenic to humans (group 2B)
- Not classifiable as to its carcinogenicity to humans (group 3)
- Probably not carcinogenic to humans (group 4)

**U.S. EPA (U.S. EPA 2003)**

- Carcinogenic to humans
- Likely to be carcinogenic to humans
- Suggestive evidence of carcinogenic potential
- Inadequate information to assess carcinogenic potential
- Not likely to be carcinogenic to humans

**U.S. NTP (NTP 2002)**

- Known to be a human carcinogen
- Reasonably anticipated to be a human carcinogen

**Cal/EPA (Cal/EPA 2004)**

- Known to the state to cause cancer.

## The Science of Carcinogen Identification and Evaluation

### The risk assessment paradigm.

**Figure 1:**
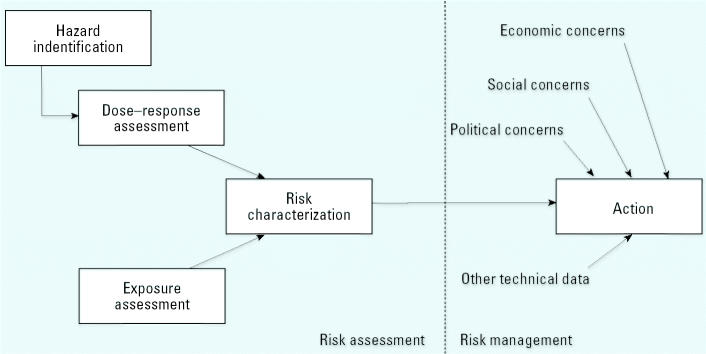
The risk assessment paradigm.

Decisions about reducing exposure to suspected carcinogens are often controversial, partly because the available data often do not allow us to identify human carcinogens with certainty and because the costs and benefits of risk reduction affect different segments of the population. In an effort to identify the scientific components of these decisions, the U.S. National Research Council (NRC) has distinguished “risk assessment,” which is the use of scientific data to describe the health effects of exposure to hazardous agents, from “risk management,” which is the process of weighing policy alternatives and selecting the most appropriate action (NRC 1983). Risk management integrates the results of a risk assessment with other technical data and with economic, social, and political concerns (Figure 1).

The NRC further divided risk assessment into a series of distinct steps (NRC 1983, 1994). Hazard identification determines whether exposure to an agent is linked to adverse health effects. Dose–response assessment characterizes the relation between the dose of an agent and the incidence of an adverse health effect. Exposure assessment determines the extent of human exposure to an agent. Risk characterization describes the nature and magnitude of human risk, including attendant uncertainty.

Under this paradigm, a cancer “hazard” is an agent that is capable of causing cancer under some circumstances, whereas a cancer “risk” is an estimate of the nature and incidence of cancer expected from a given exposure. Risk depends on both the existence of a hazard and exposure to that hazard. A cancer hazard exists even when current exposures suggest little or no cancer risk, because accidental or unanticipated exposures that are difficult to foresee may pose a risk of cancer. Thus, carcinogen identification is an exercise in hazard identification, distinct from the tasks of estimating human dose–response functions, estimating current or future human exposures, or characterizing the risk from current or future human exposures.

### Pertinent data for carcinogen identification.

The term “carcinogen” generally refers to an agent, mixture, or exposure that increases the age-specific incidence of cancer. Carcinogen identification is an activity grounded in the evaluation of the results of scientific research. Pertinent data for carcinogen identification include human epidemiologic studies, long-term bioassays in experimental animals, and other relevant data on toxicokinetics and cancer mechanisms. Each source of data has a role in the overall assessment. Epidemiologic studies can provide unequivocal evidence of a carcinogenic hazard but often are not sufficiently sensitive to identify a carcinogenic hazard except when the risk is high or involves an unusual form of cancer. In addition, cancer’s latent period implies that many years of preventable human exposure would occur before informative epidemiologic studies are available. For these reasons, animal studies generally provide the best means of assessing potential risks to humans. To answer questions about the similarity of response between animals and humans, studies of toxicokinetics and mechanisms have been employed. Toxicokinetic studies are done to allow cross-species comparisons of absorption, distribution, metabolism, and elimination but often are done in detail in only one species. Mechanistic studies aim to eventually elucidate the chemical species and cellular processes involved in cancer initiation and development.

### Evaluating evidence of cancer in humans.

Epidemiologic studies provide unique information about the response of humans exposed to potential carcinogens. Among these, cohort and case–control studies are especially useful for determining whether exposure to an agent is causally associated with human cancer. Criteria for assessing the adequacy of epidemiologic studies include selection and characterization of exposed and reference groups, identification and characterization of confounding factors and bias, duration of follow-up in view of cancer’s latent period, ascertainment of causes of disease and death, and power to detect specific effects.

In using human studies to identify carcinogens, epidemiologists often ask whether a causal interpretation is credible and whether chance, bias, or confounding factors can be excluded. On the question of causality, epidemiologists have found useful guidance in a set of factors known as the Hill criteria (Hill 1965; U.S. EPA 2003):

- Consistency of the observed association. A pattern of elevated risks observed across several independent studies would support or strengthen an inference of causality. Reproducibility of findings constitutes one of the strongest arguments for causality.
- Strength of the observed association. The finding of large, precise risks increases confidence that an association is not likely due to chance, bias, or confounding factors. A modest risk, however, does not preclude a causal association and may reflect a lower level of exposure, an agent of lower potency, or a common disease (e.g., when there is a relatively high incidence rate of a disease in the general population, it is more difficult to reach a doubling of that incidence rate).
- Specificity of the observed association. As originally described, this refers to a single cause associated with a single effect (Hill 1965). Current understanding is that many agents cause cancer at multiple sites, and many cancers have multiple causes. Thus, although the presence of specificity supports causality, its absence does not exclude it.
- Temporal relationship of the observed association. A causal interpretation is strengthened when exposure is known to precede development of the disease. Because cancer usually has a latent period ≥ 20 years, it is important to ascertain whether the study included sufficient follow-up time after exposure.
- Biologic gradient (exposure–response relationship). A clear exposure–response relationship (i.e., increasing effects associated with increasing exposure) strongly suggests cause and effect, especially when such relationships are observed for both level and duration of exposure. Because an epidemiologic study may fail to detect an exposure–response relationship for several reasons (e.g., a small range of observed exposure levels or exposure misclassification), the absence of an exposure–response relationship does not exclude a causal relationship.
- Biologic plausibility. An inference of causality is strengthened by consistency with experimental data that show plausible biologic mechanisms. A lack of mechanistic data, however, is not a reason to reject causality.
- Coherence. Other lines of evidence—for example, experimental animal studies, toxicokinetic studies, short-term tests, and mechanistic studies—may strengthen an inference of causality. The absence of other lines of evidence, however, is not a reason to reject causality.
- Experimental evidence (from human populations). Experimental evidence is seldom available from human populations and exists only when conditions of human exposure are altered to create a “natural experiment” at different levels of exposure, or for medical treatments tested in randomized controlled trials with a sufficient follow-up period. Strong evidence for causality can be provided when a change in exposure brings about a change in disease frequency, for example, the decrease in lung cancer risk that follows cessation of smoking.
- Analogy. Evidence for causality can be strengthened by information on an agent’s structural analogues.

### Evaluating evidence of cancer in experimental animals.

The most common method for identifying potentially carcinogenic agents is a long-term bioassay in experimental animals. Exposures can be tightly controlled and monitored in animal bioassays, although animal responses may not correspond strictly to human responses. Experimental carcinogenesis research is based on the scientific assumption that agents causing cancer in animals will have similar effects in humans (NTP 2002). Accordingly, in the absence of adequate data on humans, it is biologically plausible and prudent to regard agents and mixtures for which there is sufficient evidence of carcinogenicity in experimental animals as if they presented a carcinogenic risk to humans (IARC 2004).

Criteria for evaluating evidence in experimental animals include the breadth of the tumor response—for example, the induction of tumors in multiple species or in independent studies. When evaluating studies in experimental animals, it is important to incorporate scientific experience and current understanding of long-term carcinogenesis studies in laboratory animals and to consider the following points:

- Adequacy of the experimental design and conduct (e.g., route, schedule, and duration of exposure; species, strain, sex, and age of animal; duration of follow-up; tissues examined)
- Statistical significance of the observed tumor response, survival-adjusted analyses, and concerns about false positives or false negatives
- Supporting information from proliferative lesions (hyperplasia) at the site of neoplasia or in other experiments (same lesion in another sex or species)
- Progression (or lack thereof) from benign to malignant neoplasia as well as from preneoplastic to neoplastic lesions; where progression is a possibility, to assume that benign neoplasms have the potential to progress, and to combine benign and malignant tumors thought to represent stages of progression in the same organ or tissue
- Occurrence of common versus uncommon neoplasia
- Concurrent control tumor incidence, as well as the historical control rate and variability for a specific neoplasm (especially in the case of uncommon neoplasia)
- Multiplicity in site-specific neoplasia
- Latency in tumor induction
- Metastases
- Presence or absence of dose–response relationships
- Structure–activity correlations
- Genetic toxicity at the site of neoplasia.

### Evaluating mechanistic data.

Consideration of mechanistic data has the potential to improve the analysis of studies in both humans and experimental animals. Elucidation of the mechanisms of carcinogenesis can give insight into the biology of cancer and help identify stages where intervention may be possible.

In evaluating human studies, mechanistic data contribute to the discussion of biologic plausibility when evaluating whether an observed association is causal. If the series of precursor events leading to tumors in humans is understood, the observation of tumor precursors in exposed humans can provide strong support for a carcinogenic hazard.

In evaluating experimental animal studies, mechanistic studies can provide data to address the question of correspondence of response between animals and humans. This implies sufficient data to identify the mechanisms contributing to the induction of the observed animal tumors and to determine whether analogous mechanisms may be operative in humans. If this determination is based on tumor site concordance between animals and humans, it is important to discuss and document the support for this assumption, because it is not valid in general.

There has been concern recently that some assessments have been based on untested or incomplete mechanistic hypotheses. To illustrate the difficulty in drawing conclusions from mechanistic data, plausible-sounding mechanistic conclusions had been made simultaneously that 1,3-butadiene both is (Melnick and Kohn 1995) and is not (Bond et al. 1995) carcinogenic to humans. Early guidance for evaluating mechanistic data focused on the question of whether the available studies support a hypothesized sequence of precursor events [International Programme for Chemical Safety (IPCS) 1999; U.S. EPA 1999]. Patterned after the Hill criteria, these approaches looked at associations between precursor events and tumors in experimental animals.

A problem can arise, however, if insufficient consideration is given to the possibility that more than one mechanism might be operating. This can lead to premature and false conclusions, because associations observed between one mechanism’s precursor events and tumors cannot by themselves rule out the operation of other mechanisms. (This is similar to the problem of confounding factors in epidemiology: There may be strong associations between exposure and disease, but if confounding factors are not examined thoroughly, the associations may be spurious.) It is necessary to consider that an uneven level of experimental support for different mechanistic hypotheses can reflect disproportionate resources spent on investigating one hypothesis and does not exclude the contribution of other mechanisms. More recent guidance puts greater emphasis on investigating whether multiple mechanisms may be operating and asks that three questions be addressed for each potential mechanism: Is it sufficiently supported in test animals, is it relevant to humans, and which populations or life stages can be particularly susceptible? (U.S. EPA 2003).

## IARC’s Practice for Carcinogen Identification and Evaluation

The *IARC Monographs* are scientific evaluations developed by international working groups of expert scientists. These evaluations provide the scientific support for public health measures implemented by many national and international health agencies around the world. The process for developing the *IARC Monographs* is reviewed and updated from time to time. To promote better understanding of the process, here we discuss the principles and procedures currently in use.

### Selection of agents and exposures for evaluation.

Agents are selected for evaluation based on evidence of human exposure and some evidence or suspicion of carcinogenicity. Agents and exposures can be reevaluated if significant new data become available. Periodically, IARC convenes advisory groups to advise on priorities for future evaluation or reevaluation (IARC 2003). These advisory groups consist of scientists from national and international health agencies and research institutions, striving to include scientists from many countries. Seeking such advice is meant to ensure that the *IARC Monographs* reflect the current state of scientific knowledge and remain relevant to the needs of national health agencies and the research and public health communities.

### *Structure of the* IARC Monographs.

The *IARC Monographs* are published as a series of volumes. Each volume contains one or more monographs, which can cover either a single agent or a group of related agents. There is a standard structure, which has evolved to include sections on the following:

1. Exposure data

2. Studies of cancer in humans

3. Studies of cancer in experimental animals

4. Other data relevant to an evaluation of carcinogenicity and its mechanisms

5. Summary of data reported and evaluation

5.1. Exposure data

5.2. Human carcinogenicity data

5.3. Animal carcinogenicity data

5.4. Other relevant data

5.5. Evaluation

6. References.

Sections 1–4 provide a critical review of the pertinent scientific literature. Section 5 includes summaries of the scientific data and the evaluations developed by the working group.

The preamble to the *IARC Monographs* (IARC 2004) opens each volume and discusses the principles and procedures used in developing the *IARC Monographs*, including the scientific criteria that guide the working group’s evaluations. The preamble promotes consistency across different working groups and provides insight into the review process and evaluation criteria.

### The critical review of the pertinent scientific literature.

The critical review of the pertinent peer-reviewed scientific literature (sections 1–4) considers all published articles, plus articles accepted for publication. Reports and documents from national and international government agencies are considered if they are publicly available. Consensus reports in the published literature are also considered, subject to the same critical review as other articles, including consideration of the composition and balance of the panel that produced the consensus. Research that is not publicly available, including articles in preparation, is not considered.

The critical review provides a brief, separate, factual synopsis of each study, summarizing the study’s design and results. After each study synopsis is a separate assessment by the working group of the study’s strengths and limitations. These comments, which generally appear in square brackets, provide insight into the working group’s reasoning by revealing the factors that might affect their interpretation or evaluation of that study.

### The evaluations.

**Figure 2:**
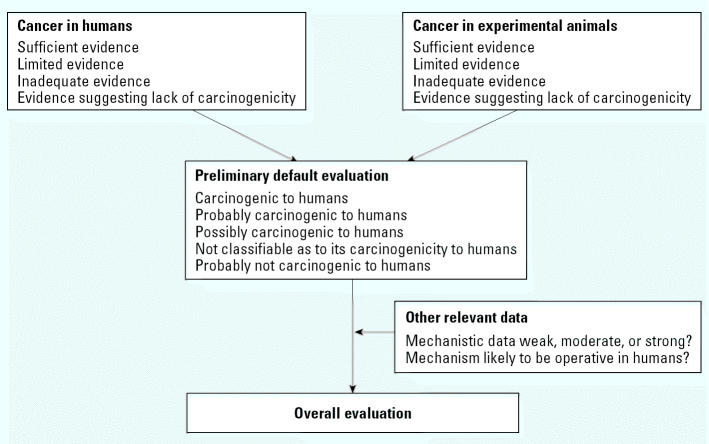
The evaluation process used in the *IARC Monographs.*

The working group develops its evaluations through a series of distinct steps (Figure 2). This stepwise evaluation process provides insight into the working group’s reasoning by revealing the weight given to each line of evidence. For each agent being evaluated, the process begins with separate evaluations of the evidence of cancer in humans and cancer in experimental animals, each choosing one of four descriptors: “sufficient evidence,” “limited evidence,” “inadequate evidence,” or “evidence suggesting lack of carcinogenicity” (for definitions of these terms, see IARC 2004).

These two partial evaluations are combined into a preliminary default evaluation that the agent is “carcinogenic to humans” (group 1), “probably carcinogenic to humans” (group 2A), “possibly carcinogenic to humans” (group 2B), “not classifiable as to its carcinogenicity to humans” (group 3), or “probably not carcinogenic to humans” (group 4). Then the mechanistic and other relevant data are considered to determine whether the default evaluation should be modified. This determination considers the strength of the mechanistic evidence and whether the mechanism operates in humans. The final overall evaluation is a matter of scientific judgment, reflecting the weight of the evidence derived from studies in humans, studies in experimental animals, and mechanistic and other relevant data. In considering all relevant scientific data, the working group may assign the agent to a higher or lower group than the default would indicate.

The goal is a consensus evaluation by the working group. The evaluation will include a synopsis that discusses the rationale for the conclusions. If the working group is not able to reach consensus, the overall evaluation is determined by majority vote. In this case, the synopsis will present the differing scientific positions, the data that support or are inconsistent with each position, and the rationale for the majority position. The evaluation can identify research needed to test different hypotheses, especially those that have not received adequate research attention.

### IARC Monograph *meetings.*

Each volume, which may contain one or more monographs, is developed by a working group at an *IARC Monograph* meeting. Each year, IARC generally convenes three separate working groups on different topics. Meetings are announced on the Internet (IARC 2004).

Before each meeting, IARC staff searches and collects the pertinent scientific literature and makes it available to the working group. Working group members critically review the literature and write first drafts of sections 1–4 on exposure, cancer in humans, cancer in experimental animals, and other relevant data, respectively. IARC collects and formats these first drafts for review at the meeting.

**Figure 3:**
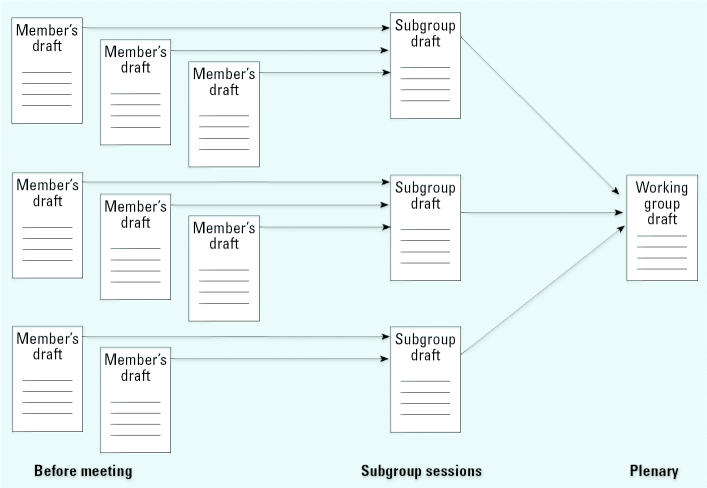
The development of drafts at *IARC Monograph* meetings.

The objectives of the meeting are review and consensus. The first days of the meeting are devoted to subgroup work. Four subgroups, each responsible for one section, peer review the individual members’ drafts, develop a joint revised draft (Figure 3), and then write the summaries that become section 5. For each agent, the subgroup on cancer in humans proposes a partial evaluation of the human evidence, and the subgroup on cancer in experimental animals proposes a partial evaluation of the animal evidence (Figure 2). The subgroup on other relevant data characterizes the mechanistic evidence using terms such as “weak,” “moderate,” or “strong” and discusses whether the mechanisms are likely to be operative in humans.

In the final days of the meeting, the subgroups come together in plenary session. The entire working group peer reviews and reaches consensus on the critical reviews in sections 1–4 and discusses and reaches consensus on the summaries and partial evaluations proposed by the subgroups. Then the working group as a whole develops and reaches consensus on an overall evaluation of each agent.

### *Declaration of interests by participants at* IARC Monograph *meetings.*

IARC, part of the WHO, follows WHO procedures with respect to declaration of interests by participants in its meetings (WHO 2004). Each potential participant is asked to declare, in confidence,

any interests that could constitute a real, potential or apparent conflict of interest, with respect to his/her involvement in the meeting or work, between *a*) commercial entities and the participant personally, and *b*) commercial entities and the administrative unit with which the participant has an employment relationship.

The WHO defines conflict of interest to mean “the expert or his/her partner, or the administrative unit with which the expert has an employment relationship, has a financial or other interest that could unduly influence the expert’s position with respect to the subject-matter being considered.” An apparent conflict of interest exists when “an interest would not necessarily influence the expert but could result in the expert’s objectivity being questioned by others” (WHO 2004).

The WHO provides several examples of financial or other interests, including competing interests, that should be declared. The examples include consulting work or research support that can pose as much of a conflict as employment or stock ownership. In addition, a conflict can arise from an expectation of future support, to the expert individually or to the expert’s organization. On the other hand, an interest that is no longer current becomes immaterial after a period of time. In the case of research support given to an expert’s organization, determining whether the conflict warrants some limitation on participation includes consideration of several factors, such as the level of funding from interested parties, whether the organization’s research or positions depend on such funding, and whether such funding supports the expert’s own research or position.

Before an invitation is extended, each potential participant submits a declaration of interests (WHO 2004). IARC assesses these interests to determine whether there is a conflict that warrants some limitation on participation. Each participant updates the declaration of interests at the opening of the meeting. Interests pertinent to the subject matter of the meeting are disclosed to the meeting participants and in the published *IARC Monograph.*

### *Participants in* IARC Monograph *meetings*.

Two principles govern the selection of working group members: to invite the best-qualified experts, and to avoid real or apparent conflicts of interest. Consideration is given also to demographic diversity. Members are chosen on the basis of knowledge and experience, which can come from research into the specific agents to be evaluated or from general experience in conducting or evaluating epidemiologic or experimental studies. Members chair the meeting and the subgroups and are the only participants who vote on the overall evaluations, if a vote is needed. Working group members are invited to serve in their individual capacities as scientists and not as representatives of their government or any organization with which they are affiliated.

A difficulty arises when an expert with relevant knowledge and experience has a real or apparent conflict of interest. This issue has become more visible in recent years because commercial interests sponsor many epidemiologic and experimental studies, and some investigators develop a history of receiving research support from interested parties. The selection of experts with real or apparent conflicts of interest can erode confidence in the integrity and impartiality of the results. This creates a tension between two competing ideals: evaluations developed by the best-qualified experts versus evaluations whose integrity and impartiality are above question.

The new category of invited specialist allows the *IARC Monographs* to achieve both ideals. An invited specialist is an expert with critical knowledge and experience who is recused from certain activities because of a real or apparent conflict of interests. These activities include serving as meeting chair or subgroup chair, drafting text that discusses cancer data or contributes to the evaluations (sections 2–4 and 5.2–5.5), and participating in evaluations reached by either consensus or vote. Invited specialists are available during subgroup and plenary discussions to contribute the benefit of their knowledge and experience. Invited specialists also agree to serve in their individual capacities as scientists and not as representatives of any organization or interest. Their conflicting interests are fully disclosed to the meeting participants and in the *IARC Monograph*. In this way, the meeting can include the best-qualified experts, and the evaluations are developed and written by experts with no real or apparent conflicts of interest.

In the interest of transparency, a limited number of scientifically qualified observers are welcome to attend *IARC Monograph* meetings. Consideration is given to admitting observers from different constituencies with differing interests. The main role of observers is to serve as sources of first-hand information from the meeting to the organizations that sponsor them. Observers can play a valuable role in ensuring that all published information and scientific perspectives are considered. At the meeting, the meeting chair and subgroup chairs may grant observers the opportunity to speak. Observers do not serve as meeting chair or subgroup chair, draft any part of an *IARC Monograph*, or participate in the evaluations. Observers may be presumed to serve the interests of the organizations that nominate and sponsor them, and these interests are fully disclosed to the meeting participants and in the *IARC Monograph*. Observers are asked to agree to ethics guidelines that include a requirement not to lobby working group members, both before and during the meeting. A challenge for IARC is to increase the diversity of observers in view of the unequal resources available to potential observers from different sectors.

There are two other categories of participants. Representatives of national and international health agencies (e.g., the U.S. National Cancer Institute) often attend and provide independent assurance and guarantee of the integrity of the *IARC Monographs*. Scientists employed by IARC comprise the IARC Secretariat. The secretariat hosts the meeting and drafts text or tables when requested by the meeting chair or subgroup chair. To facilitate consistency across different *IARC Monographs*, members of the secretariat serve as rapporteurs and answer questions about the preamble. After the meeting, the secretariat reviews all data cited in the text and tables to ensure scientific accuracy and clarity and publishes the finished volume. Representatives and secretariat participate in discussions but do not vote on the evaluations; thus, the evaluations are determined by working group members only.

Table 1 summarizes the roles of working group members, invited specialists, observers, representatives, and the secretariat. The published volume identifies all participants by name and affiliation and identifies the meeting chair and subgroup chairs.

### Inclusion of all scientific views.

When planning a meeting, it is important to identify the pivotal issues in advance and convene a working group that includes all scientific views. There are two reasons for this. First, a balanced representation of all scientific views promotes confidence that all hypotheses and data have been considered fully and evenly. Second, identifying the pivotal issues can uncover issue-related conflicts that would not otherwise be apparent but may warrant some limitation on participation. For example, the pivotal issue of whether a particular mechanism is operative in humans not only affects the evaluation of the agent being considered but also can set a precedent for other agents that operate through similar mechanisms. Identifying pivotal issues and related agents can be difficult, but doing so will promote confidence in the working group’s objectivity.

### Freedom from interference.

The working group should be free from all attempts at interference, before and during the meeting. This includes lobbying by interested parties, receipt of written materials from interested parties, and meals, drinks, social events, or other favors offered by interested parties. Attempts at interference outside the meeting are particularly insidious, because they occur outside the view of other participants. Such interference destroys transparency and invites suspicion. Working group members have assumed the responsibility to safeguard the integrity of their work by resisting any attempt at interference. To aid them in this responsibility, working group members are reminded not to discuss the subject matter of the meeting with those outside the meeting and are asked to report all attempts at interference.

## The Future of Carcinogen Identification and Evaluation

The future of carcinogen identification will be one of continuing evolution to reflect changes in the underlying science. Future evaluations will continue to consider mechanistic data to aid in interpreting experimental animal results. The task will be not only to get the right answer based on publicly available scientific evidence, but also to build a broad-based scientific consensus around the answer. When sufficient data are available to identify a mechanism of carcinogenesis, these data will also be the key to identifying susceptible populations and life stages, including the prenatal and early postnatal periods. Another implication of using mechanistic data will be carcinogen identifications that are based on scientific inference in the absence of tumor studies in humans or experimental animals.

In addition to changes in the science, the milieu in which carcinogens are identified is changing rapidly. A key challenge is to maintain independence against the increasing demand for access and influence by advocates on all sides. Another is keeping current, as more agents need evaluation because of new scientific data or understanding.

In its practice of carcinogen identification, IARC is committed to the highest standards of scientific and ethical conduct. For > 30 years the *IARC Monographs* have achieved a reputation unmatched for thoroughness, accuracy, and integrity. The principles and procedures discussed here should ensure that this reputation remains solid well into the future.

## Figures and Tables

**Table 1:** Roles of participants at *IARC Monograph* meetings.

	Working group members	Invited specialists	Observers	Representatives	IARC secretariat
Before the meeting					
Draft section 1 (exposure data)	×	×			×
Draft sections 2–4	×				×
During subgroup sessions					
Serve as subgroup chair	×				
Peer review members’ drafts (sections 1–4)	×	×		×	×
Draft summary of section 1 (section 5.1)	×	×			×
Draft summaries of sections 2–4 (sections 5.2–5.4)	×				×
Propose evaluations of human, animal, or mechanistic data	×				
During plenary session					
Serve as meeting chair	×				
Peer review subgroup drafts and summaries	×	×		×	×
Discuss subgroup evaluations and develop the overall evaluation (section 5.5)	×			×	×
Vote on overall evaluation, if needed	×				
